# Turner Syndrome and Associated Problems in Turkish Children: A Multicenter Study

**DOI:** 10.4274/jcrpe.1771

**Published:** 2015-03-05

**Authors:** Ediz Yeşilkaya, Abdullah Bereket, Feyza Darendeliler, Firdevs Baş, Şükran Poyrazoğlu, Banu Küçükemre Aydın, Şükran Darcan, Bumin Dündar, Muammer Büyükinan, Cengiz Kara, Erkan Sarı, Erdal Adal, Ayşehan Akıncı, Mehmet Emre Atabek, Fatma Demirel, Nurullah Çelik, Behzat Özkan, Bayram Özhan, Zerrin Orbak, Betül Ersoy, Murat Doğan, Ali Ataş, Serap Turan, Damla Gökşen, Ömer Tarım, Bilgin Yüksel, Oya Ercan, Şükrü Hatun, Enver Şimşek, Ayşenur Ökten, Ayhan Abacı, Hakan Döneray, Mehmet Nuri Özbek, Mehmet Keskin, Hasan Önal, Nesibe Akyürek, Kezban Bulan, Derya Tepe, Hamdi Cihan Emeksiz, Korcan Demir, Deniz Kızılay, Ali Kemal Topaloğlu, Erdal Eren, Samim Özen, Saygın Abalı, Leyla Akın, Beray Selver Eklioğlu, Sultan Kaba, Ahmet Anık, Serpil Baş, Tolga Ünüvar, Halil Sağlam, Semih Bolu, Tolga Özgen, Durmuş Doğan, Esra Deniz Çakır, Yaşar Şen, Nesibe Andıran, Filiz Çizmecioğlu, Olcay Evliyaoğlu, Gülay Karagüzel, Özgür Pirgon, Gönül Çatlı, Hatice Dilek Can, Fatih Gürbüz, Çiğdem Binay, Veysel Nijat Baş, Kürşat Fidancı, Adem Polat, Davut Gül, Cengizhan Açıkel, Hüseyin Demirbilek, Peyami Cinaz, Carolyn Bondy

**Affiliations:** 1 Gülhane Military Medicine Academy, Department of Pediatric Endocrinology, Ankara, Turkey; 2 Marmara University Faculty of Medicine, Department of Pediatric Endocrinology, İstanbul, Turkey; 3 İstanbul University Istanbul Faculty of Medicine, Department of Pediatric Endocrinology, İstanbul, Turkey; 4 Ege University Faculty of Medicine, Department of Pediatric Endocrinology, İzmir, Turkey; 5 Katip Çelebi University Faculty of Medicine, Department of Pediatric Endocrinology, İzmir, Turkey; 6 Konya Training and Research Hospital, Clinic of Pediatric Endocrinology, Konya, Turkey; 7 Ondokuz Mayıs University Faculty of Medicine, Department of Pediatric Endocrinology, Samsun, Turkey; 8 Kanuni Sultan Süleyman Training and Research Hospital, Clinic of Pediatric Endocrinology, İstanbul, Turkey; 9 Inönü University Faculty of Medicine, Department of Pediatric Endocrinology, Malatya, Turkey; 10 Necmettin Erbakan University Faculty of Medicine, Department of Pediatric Endocrinology, Konya, Turkey; 11 Yıldırım Beyazıt University Faculty of Medicine, Department of Pediatric Endocrinology, Ankara, Turkey; 12 Gazi University Faculty of Medicine, Department of Pediatric Endocrinology, Ankara, Turkey; 13 Dr. Behçet Uz Children Hospital, Clinic of Pediatric Endocrinology, İzmir, Turkey; 14 Pamukkale University Faculty of Medicine, Department of Pediatric Endocrinology, Denizli, Turkey; 15 Atatürk University Faculty of Medicine, Department of Pediatric Endocrinology, Erzurum, Turkey; 16 Celal Bayar University Faculty of Medicine, Department of Pediatric Endocrinology, Manisa, Turkey; 17 Yüzüncü Yıl University Faculty of Medicine, Department of Pediatric Endocrinology, Van, Turkey; 18 Harran University Faculty of Medicine, Department of Pediatric Endocrinology, Şanlıurfa, Turkey; 19 Uludağ University Faculty of Medicine, Department of Pediatric Endocrinology, Bursa, Turkey; 20 Çukurova University Faculty of Medicine, Department of Pediatric Endocrinology, Adana, Turkey; 21 İstanbul University Cerrahpaşa Faculty of Medicine, Department of Pediatric Endocrinology, İstanbul, Turkey; 22 Kocaeli University Faculty of Medicine, Department of Pediatric Endocrinology, Kocaeli, Turkey; 23 Osmangazi University Faculty of Medicine, Department of Pediatric Endocrinology, Eskişehir, Turkey; 24 Karadeniz Technical University Faculty of Medicine, Department of Pediatric Endocrinology, Trabzon, Turkey; 25 Dokuz Eylül University Faculty of Medicine, Department of Pediatric Endocrinology, İzmir, Turkey; 26 Diyarbakır Children State Hospital, Clinic of Pediatric Endocrinology, Diyarbakır, Turkey; 27 Gaziantep University Faculty of Medicine, Department of Pediatric Endocrinology, Gaziantep, Turkey; 28 Düzce University Faculty of Medicine, Department of Pediatric Endocrinology, Düzce, Turkey; 29 Selçuk University Faculty of Medicine, Department of Pediatric Endocrinology, Konya, Turkey; 30 Keçiören Training and Research Hospital, Clinic of Pediatric Endocrinology, Ankara, Turkey; 31 Süleyman Demirel University Faculty of Medicine, Department of Pediatric Endocrinology, Isparta, Turkey; 32 Kayseri Training and Research Hospital, Clinic of Pediatric Endocrinology, Kayseri, Turkey; 33 National Institute of Child Health and Human Development, Bethesda, Maryland, USA

**Keywords:** Nationwide study, Turner syndrome, children, diagnostic features, associated problems

## Abstract

**Objective:**

Turner syndrome (TS) is a chromosomal disorder caused by complete or partial X chromosome monosomy that manifests various clinical features depending on the karyotype and on the genetic background of affected girls. This study aimed to systematically investigate the key clinical features of TS in relationship to karyotype in a large pediatric Turkish patient population.

**Methods:**

Our retrospective study included 842 karyotype-proven TS patients aged 0-18 years who were evaluated in 35 different centers in Turkey in the years 2013-2014.

**Results:**

The most common karyotype was 45,X (50.7%), followed by 45,X/46,XX (10.8%), 46,X,i(Xq) (10.1%) and 45,X/46,X,i(Xq) (9.5%). Mean age at diagnosis was 10.2±4.4 years. The most common presenting complaints were short stature and delayed puberty. Among patients diagnosed before age one year, the ratio of karyotype 45,X was significantly higher than that of other karyotype groups. Cardiac defects (bicuspid aortic valve, coarctation of the aorta and aortic stenosis) were the most common congenital anomalies, occurring in 25% of the TS cases. This was followed by urinary system anomalies (horseshoe kidney, double collector duct system and renal rotation) detected in 16.3%. Hashimoto’s thyroiditis was found in 11.1% of patients, gastrointestinal abnormalities in 8.9%, ear nose and throat problems in 22.6%, dermatologic problems in 21.8% and osteoporosis in 15.3%. Learning difficulties and/or psychosocial problems were encountered in 39.1%. Insulin resistance and impaired fasting glucose were detected in 3.4% and 2.2%, respectively. Dyslipidemia prevalence was 11.4%.

**Conclusion:**

This comprehensive study systematically evaluated the largest group of karyotype-proven TS girls to date. The karyotype distribution, congenital anomaly and comorbidity profile closely parallel that from other countries and support the need for close medical surveillance of these complex patients throughout their lifespan.

## INTRODUCTION

Turner syndrome (TS) is a genetic disorder characterized by total or partial absence of one sex chromosome (1). It is one of the most common chromosomal disorder with an incidence of 1:2500 female live births (2,3). The most prevalent karyotype is 45,X, followed by mosaic patterns.

TS is associated with several morbidities that increase with age. Although TS causes several multisystem disorders, the most common presentation is usually due to short stature and primary gonadal deficiency. Patients may present with congenital malformations such as horseshoe kidney and coarctation of the aorta. They may also develop diabetes, hypothyroidism, hypertension, hearing loss, osteoporosis and bone fractures (4,5). Timely diagnosis and proper management of associated problems may reduce substantial morbidity and mortality and improve the quality of life in TS patients. Studies based on a large pediatric population which provide data on frequency of associated problems and their distribution among the different karyotypes are scarce (6,7,8).

In the present study, we aimed to define the frequency of associated problems in TS patients during childhood and the distribution of these clinical features according to karyotype and age by evaluating 842 patients with TS from 35 centers in Turkey.

## METHODS

### Patients and Data Collection

In this study, we retrospectively evaluated the files of 842 TS patients aged 0-18 years who were being followed in 35 different centers from different regions of Turkey between September 2013 and February 2014. The study was approved by the Ethics Committee of Gülhane Military Medical Academy. All collected data were obtained from patient hospital records; no private information was included in the study.

A common case recording form (CRF) was created after literature review by four physicians (EY, FD, ES and PC) who were all experienced in TS and an expert on electronic CRF preparation (CA). The CRF covered demographic features, as well as clinical and laboratory findings of TS patients. The CRF was uploaded to the website of FAVOR Web Registry System (www.favorsci.org). Data entered in the registry were also checked for consistency by a research assistant (EY).

Physicians who were conducting an outpatient clinic for TS patients were asked to register their TS patients using the paper-based study forms. Another physician in each center was asked to register each patient online using the FAVOR Registry System. The time given for patient enrollment was four months and this time period ended on January 31, 2014, after which date the collected patient record data were entered to Microsoft Excel database and subsequently transferred to SPSS for Windows statistical software for statistical analysis.

### Clinical and Laboratory Evaluation

The karyotype and the presence of sex determinant region of Y (SRY) for all patients were questioned. A standard 30-cell karyotype analysis from peripheral blood was made. Fluorescent in situ hybridization (FISH) was performed in case of strong suspicion of undetected mosaicism and 125/842 patients were analyzed for SRY by FISH method. Some centers screened all patients for SRY, but SRY results were not available from all centers. Birth history and presenting characteristics (reason for admission, age of admission at presentation) were recorded. Findings on detailed questions relating to the cardiac, renal, gastrointestinal, endocrine systems, ear nose and throat, eye, skin and skeletal system, as well as psychosocial and metabolic problems that were relevant to TS were recorded by the physicians. Cardiac and renal findings were based on ultrasound examinations. Thyroid diseases were evaluated by testing for anti-thyroid peroxidase antibody, anti-thyroglobulin antibody, thyroid function tests and thyroid sonography.

Karyotypes of patients were classified according to numerical, structural and both numerical and structural abnormalities by a geneticist (DG). Patients with a body mass index (BMI) between 85-95 percent were defined as overweight and patients with a BMI over 95 percent as obese (9). Insulin resistance was assessed according to Turkish standards. Homeostasis model assessment of insulin resistance cut-off values in girls were calculated to be 2.22 in the pre-pubertal period and 3.82 in the pubertal period (10). Dyslipidemia was evaluated according to the recommendations of Integrated Guidelines for Cardiovascular Health and Risk Reduction in Children and Adolescents (11). A bone mineral density (BMD) Z-score less than -2.5 was defined as osteoporosis and a BMD Z-score between -1 and -2.5 as osteopenia (12). BMD was measured by dual-energy X-ray absorptiometry and was corrected for stature.

### Statistical Analysis

Statistical analyses were performed using SPSS for Windows V. 22.0 statistical software. Frequencies and percentages represented the descriptive statistics for categorical variables and mean ± standard deviation values were used for continuous variables. Ratios were compared using chi-square test and mean values were compared using t-test. A p-value less than 0.05 was considered as statistically significant.

## RESULTS

A total of 842 patients with TS were registered. The distribution of patients according to karyotype analysis was as follows: 45,X (n=427; 50.7%), 45,X/46,XX (n=91; 10.8%), 46,X,i(Xq) (n=85; 10.1%) and 45,X/46,X,i(Xq) (n=82, 9.5%) (Table 1). The number of patients showing a mosaic pattern was 294 (34.9%), 297 patients (35.3%) had X chromosome structural abnormalities and 167 patients (19.8%) had isochromosome X. Among the study cohort, the number of girls with a Y chromosome in karyotyping was 34 (4%).

SRY analysis was performed in 106 patients. Five of the patients (3 patients with 45,X karyotype, 1 with 46,Xi(Xq) and another with 45,X/46,X(Xp) were found to be positive for SRY among these 106 patients who were not detected to have a Y chromosome material by using standard methodology.

**Figure 1:**
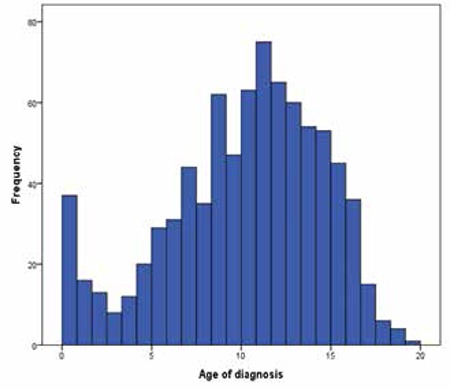
Histogram of age at diagnosis in children with Turner syndrome

The mean gestational age, birth weight and height of the patients were 39±2 weeks, 3000±580 g and 48.0±2.5 cm, respectively. The mean age of presentation to clinics was 10.2±4.4 years for the whole group. It was 10.1±4.8 years for patients with 45,X karyotype and 10.3±4.0 years for the rest of the karyotypes, p=0.391). Age at diagnosis was between the ages 1 and 12 years in 57.1% of the patients and older than 12 years in 37.2%. A histogram according to age at diagnosis is given in Figure 1. About 5.7% of the patients were diagnosed before one year of age. Among patients diagnosed before age one year, the ratio of karyotype 45,X (n=29; 72.5%) was significantly higher than that of mosaic karyotype (n=6, 20%), structural (n=7; 17.5%) and isochromosome abnormalities (n=3; 7.5%). In patients diagnosed under 5 years of age, 66 out of 105 (62.9%) had 45,X karyotype.

The most frequent complaints at presentation were short stature in 708 (84.1%) patients, delayed puberty in 130 (15.4%) patients and edema in 37 (4.4%). Other presenting complaints in 128 (15.2%) patients were dysmorphism, congenital heart diseases and antenatal diagnosis. Mean ages of presentation for short stature and delayed puberty were 10.0±3.3 years and 16.4±2.6 years, respectively.

The number of patients who were investigated for associated problems and frequencies of pathologies detected in the registered 842 TS patients are presented below.

### Cardiovascular Pathologies

Cardiac evaluation was performed by transthoracic echocardiography in 719 patients. Cardiovascular pathologies were detected in 180 (25%). Bicuspid aortic valve (n=61; 8.6%), coarctation of the aorta (n=46; 6.5%) and aortic stenosis (n=38; 5.4%) were the most frequently detected abnormalities (Table 2). Seventeen patients had mitral insufficiency and 11 had mitral valve prolapse. Ten patients had dilatation of the aorta and transthoracic echocardiography findings showed that 16 patients had regurgitation of the aortic valve. Fifteen patients had hypertension. Forty-two cases presented with other cardiac abnormalities [atrial septal defect (14 cases), patent foramen ovale (6 cases), pulmonary stenosis (6 cases), ventricular septal defect (5 cases), partial anomalous pulmonary venous return (3 cases), others (8 cases)]. Cardiac malformations were more common in patients with 45,X karyotype (21.7%) compared to X mosaicism (13.6%), X chromosome structural abnormalities (9.9%) and isochromosome (9.3%). Coarctation of the aorta was more common in patients with 45,X karyotype (10.8%) compared to those with X chromosome structural abnormalities (2.1%), isochromosome (1.4%) and X mosaicism (1.2%).

### Renal Pathologies

A total 117 out of 714 (16.3%) patients had renal anomalies detected on ultrasound screening. The most frequent ones were horseshoe kidney (9.0%) followed by double collector duct system (2.7%) and rotation anomalies (1.3%). Other encountered anomalies (renal agenesis, renal ectopia, etc) are listed in Table 2. We did not establish any difference between karyotypes with regard to renal anomalies.

### Thyroid Pathologies

Findings indicative of Hashimoto’s or Graves diseases were evaluated in 792 patients by testing for anti-thyroid peroxidase antibody, anti-thyroglobulin antibody, free thyroxine (T4), thyroid stimulating hormone (TSH) and/or TSH receptor antibodies and/or thyroid sonography. 74 patients were found to have hypothyroidism and 14 patients subclinical hypothyroidism with high TSH but normal free T4 levels. Thyroid autoantibodies were positive in 82 out of 792 (10.4%) patients of which 42 (51.2%) had hypothyroidism. 74% of the patients with positive thyroid autoantibody were older than 10 years, 24% of them were between 5-10 years and 2% were below 5 years of age. Only 3 patients had Graves disease. Thyroid autoimmunity was more common in patients with isochromosome karyotype (16.7%) and its frequency was significantly higher than that of other karyotype groups (9.2%) (p=0.008). No difference was observed among karyotypes with regard to hypothyroidism.

### Gastrointestinal Pathologies

Gastrointestinal abnormalities were evaluated in 698 patients by testing for liver function tests and celiac disease screening (testing for anti-transglutaminase, anti-gliadin and anti-endomysium antibodies). Endoscopy, gastroscopy, colonoscopy and biopsy were performed in suspected cases. Gastrointestinal abnormalities were detected in 62 patients (8.9%). Transaminases were elevated in 24 patients and 9 patients had hepatosteatosis. Thirty-two patients were positive for celiac antibodies. The diagnosis of celiac disease had been confirmed by endoscopy in 18 patients. The most frequent karyotypes among 9 patients with hepatosteatosis was isochromosome X (p=0.008) and structural abnormalities (p=0.005).

### Ear, Nose and Throat Problems

Evaluation for ear, nose and throat problems showed pathology in 122 out of 539 (22.6%) TS patients. Recurrent otitis media was the most common finding with 77 patients (14.3%), followed by hearing loss in 54 (10.0%) patients of whom 44 had conductive and 10 had sensory-neural type hearing loss. We did not find any correlation between recurrent otitis media and conductive hearing loss. Nineteen patients had undergone tympanostomia. Ear problem frequencies did not differ among various karyotypes.

### Ophthalmologic Problems

Although we do not know the number of patients who had been evaluated, ophthalmologic problems were reported to be detected in 74 patients, most of which were strabismus encountered in 41, myopia in 16 and ptosis in 8 patients.

### Dermatological Problems

Dermatological problems were detected in 159 out of 729 (21.8%) patients. The most prevalent dermatologic problem was presence of nevi, observed in 114 (15.6%) patients. Psoriasis, vitiligo, alopecia and keloid were other reported dermatological problems. A statistically significant increase was detected in the frequency of nevus by age (p=0.001). The frequency of nevi was 21.8% in patients older than 10 years, 11.6% in those between 5-10 years and 4.8% in those under 5 years of age. Frequency of dermatological problems did not vary among different karyotype groups.

### Orthopedic and Metabolic Bone Problems

Orthopedic evaluation by an orthopedist and/or DEXA measurement was made in 442 patients and 122 (27.6%) were found to have at least one problem. Osteoporosis and osteopenia were the most common findings in 63 (15.3%) and 49 (11.1%) patients, respectively. Scoliosis and hip dislocation were some other reported pathologies.

### Psychosocial Problems

Two hundred and ninety-one patients were assessed for psychosocial problems by pediatric and adolescent psychiatrists and/or were reported to have undergone such an assessment in their school files. The most common problems were learning difficulty (16.1%) as well as vague mental (13.7%) and emotional (6.9%) problems (Table 2).

### Metabolic Problems

Metabolic evaluation was done in 727 patients; 69 (9.5%) of these were obese and 40 (5.5%) were overweight. Insulin resistance (p=0.042) and glucose intolerance (p=0.003) were more common in patients with isochromosome X, but there was no association with obesity. Only 3 patients had type 2 diabetes and 1 patient had type 1 diabetes. The metabolic test results of TS patients of normal weight and those of overweight or obese TS patients are presented in Table 3. Among 711 patients evaluated for dyslipidemia, 81 (11.4%) had the disease. The number of patients with high total cholesterol levels was 48 (6.8%), the number of patients with high triglyceride levels was 36 (5.1%) and the number of patients with high LDL levels was 27 (3.8%).

## DISCUSSION

In this study, we reviewed the presenting complaints, clinical characteristics, associated disorders and distribution of these according to karyotype analysis in 842 patients with TS from 35 centers in Turkey.

TS patients mostly present with X chromosome monosomy, followed by mosaic pattern. The most common forms of mosaicism are reported as 45,X/46,XX and 45,X/46,X,i(Xq) and the most commonly reported X chromosome structural abnormality is isochromosome Xq (1,2,3). In our population, the frequency of 45,X monosomy was 50.7% and that of mosaicism was 35.4%. The most frequent mosaic patterns were 45,X/46,XX (10.8%) and 45,X/46,X,i(Xq) (9.5%). Among structural abnormalities, pure isochromosome Xq structural abnormality was the most prevalent (10.1%). These findings are in agreement with the literature.

Identification of Y chromosome material in females with TS is important because of the risk of gonadoblastoma. Mosaicism for a cell line with a normal or abnormal Y chromosome is identified in 6% to 11% of patients with TS with standard cytogenetic techniques (13). Occult Y chromosome mosaicism detected by techniques other than standard cytogenetic in TS is variable in the different studies and also varies with the methodology used. In a meta-analysis of studies on a total of 541 patients with TS without Y chromosome material on routine cytogenetic analysis, frequency of mosaicism for a Y-containing cell line using molecular techniques (Southern blot and/or polymerase chain reaction) was reported to be 5%.

The percentage of patients with Y chromosome mosaicism (by molecular or standard cytogenetic techniques) was 8%; of these, 12% had gonadoblastoma. Detection of occult Y mosaicism in 45,X subjects using interphase FISH with a probe for the Y centromere (DYZ3) has been reported to range from 0% to 4% (14,15). In our cohort, while 4% out of 842 cases were defined to have Y material using the standard method, 5/106 (4.7%) of SRY negative patients by the standard method were found to be SRY positive using the FISH method.

The diagnosis of TS is usually delayed. In up to 10% of patients, diagnosis may be delayed until adulthood (1). In our study, the mean age at diagnosis was 10.2±4.4 years. The most common complaint at the first presentation is short stature and this is followed by a complaint of delay in puberty (2,4). In 70% of our cases, short stature was the presenting complaint and in line with the literature, the majority of our patients (86.3%) had short stature and/or delay in puberty. Patients with karyotype 45,X or isochromosome Xq were diagnosed at an earlier age than those with other karyotypes. Frequency of diagnosis before age 5 years was 15.7% in patients with 45,X. This frequency was 9.0%, 9.1% and 9.7% in TS patients with mosaic, isochromosome and structural abnormalities, respectively.

TS is associated with a wide range of abnormalities affecting nearly every organ system. Congenital cardiovascular defects constitute the most important life-threatening pathology in these patients. It has been reported that the 50% of adult patients and 30% of pediatric patients with TS present with cardiovascular abnormalities (16,17). In our study, the frequency of cardiac abnormality in TS patients aged between 0-18 years was 25%, a finding consistent with previous reports (18,19). A bicuspid aortic valve is reported as the most frequent congenital cardiac defect in TS (11-16%). A bicuspid aortic valve is reported in only 1-2% of the general population. It is usually an isolated abnormality. Next in frequency is coarctation of the aorta (7-11%) that is an important cause of hypertension and aortic stenosis/insufficiency (3-5%). Other cardiac pathologies include structural defects such as partial anomalous pulmonary venous connection, atrial and ventricular septal defects (18,19,20,21). In the present study, we found a smaller proportion of patients with bicuspid aortic valve (8.6%) compared to previous reports, but the frequencies of coarctation of the aorta (6.5%) and aortic stenosis (5.4%) were consistent with the literature.

In studies with large number of patients, it was also found that cardiac malformations were more prevalent among patients with 45,X than among patients with X mosaicism and X chromosome structural abnormalities. Similarly, coarctation of the aorta predominates in patients with the karyotype of 45,X (22,23,24,25,26,27). In our study also, cardiac malformations were significantly more common in patients with 45,X karyotype (21.7%) compared to other karyotypes (12.3%, p=0.001). Frequency of coarctation of the aorta was 6.5% and most of these patients showed a 45,X karyotype (only 3 patients with coarctation of aorta had mosaicism). Some congenital cardiovascular anomalies may not have been detected using conventional imaging techniques. This is especially true for X mosaicism which includes 45X/46XX and 45X/46XdefectX. Because the former may have a milder phenotype, the latter are fully affected as all cells are missing the X (or Y) chromosome short arm (1,2).

Hypertension has been reported in 7-17% of children and 24-40% of adults with TS (2,28,29,30,31). Possible mechanisms involved in the development of hypertension in this group of patients are abnormal vasculature, obesity, obstructive sleep apnea and metabolic syndrome. It has been stated that conventional blood pressure measurements may lead to underdiagnosis of hypertension in individuals with TS and ambulatory blood pressure monitoring is recommended. The frequency of hypertension in our study was 2.1%, a lower rate than in previous reports. This low frequency was thought to be due to a low rate of use of ambulatory blood pressure measurement method.

Renal pathologies have been reported to be of an approximate frequency of 30% in TS patients, a frequency which is nine times greater compared to the normal population (1,32,33,34). Common renal anomalies are horseshoe kidney, double collecting duct systems or rotational abnormalities each with a 5% to 10% frequency (2). In our study, renal abnormality rate was 16.3% and these were mostly horseshoe kidney (9.0%), double collecting duct systems (2.7%) and rotation problems (1.3%). Although there are different findings in the literature about karyotype and renal abnormalities (35), our findings did not demonstrate any karyotype differences with regard to renal abnormalities.

TS patients have an increased risk of thyroid diseases (thyroiditis, hypothyroidism, etc.) (4). In the present study, we found that 88 out of 792 (11.1%) TS patients suffered from hypothyroidism, either overt or subclinical. Antibody positivity was 10.7%. Radetti et al (36) studied 478 TS females with a mean age of 15.5 years and found that 22.2% had positive thyroid autoantibodies. Compensated hypothyroidism and hypothyroidism were present in 6.1% of these patients. In TS patients, thyroid dysfunction and autoimmunity increase with age (37). A study investigating TS patients younger than 30 years reported that thyroid autoantibody was positive in 16.7% of patients between 0-9.9 years, 47.7% between 10-19.9 years and 37.5% between 20-29.9 years of age (38). In our study also, a striking increase was observed in the frequency of thyroid autoantibody positivity by age; 74% of the patients with positive thyroid autoantibodies were over 10 years, 24% of them were between 5-9.99 years and 2% were younger than 5 years. We observed that antibody positivity significantly increased with age. Autoimmune thyroid disease has been found to be mostly prevalent in women with the isochromosome [46,Xi(Xq)] karyotype than other karyotypes (39,40). Similarly, in our study, a positive thyroid autoantibody was found to be more prevalent among patients with isochromosome X compared to other groups (p=0.008). No difference was observed among karyotypes regarding hypothyroidism.

Elevated liver enzymes and increased risk of cirrhosis have been reported in patients with TS (41). Improvement in liver functions after estrogen replacement suggested the role of estrogen deficiency (42). In the present study, 62 out of 698 patients (8.9%) had gastrointestinal pathologies. Transaminases were elevated in 24 (3.4%) patients, of whom 5 were younger than 10 years of age. Nine (1.3%) patients had hepatosteatosis and 4 of these patients were non-obese.

Patients with TS have a 2-10 fold increased risk of developing inflammatory bowel disease (3%) and a 11-fold increased risk of developing (2.2-8.1%) celiac disease as compared to the normal population (2,29,43,44). Inflammatory bowel disease was not detected in this present study. Biopsy-proven celiac disease was detected in 18 (2.5%) and celiac antibody positivity in 32 (4.5%) patients. Prevalence of celiac disease in our cohort was higher than in healthy Turkish school children which have a reported prevalence of 0.47% (45). No karyotypic characteristics were observed in the 6 of the 18 celiac patients investigated.

Craniofacial malformations that cause Eustachian tubes dysfunction may result in recurrent otitis media and consequent hearing problems in children with TS. Hearing problems may be seen up to 30% in childhood and up to 90% in adulthood in patients with TS (46,47). Conductive type hearing loss is mostly prevalent in early ages, while sensorineural hearing loss generally develops in late childhood or early adulthood and shows an increase with age (7,48,49). In our cohort, 122 (22.6%) patients had ear, nose and throat problems. Recurrent otitis media was the most common finding and existed in 77 patients. Hearing loss was detected in 54 patients; 44 of these had conductive type and 10 sensorineural type hearing loss. Hearing loss was more prevalent among patients older than 5 years. Hearing problems are reported to be least prevalent in TS patients with mosaicism and more severe in patients with 45,X monosomy, isochromosome X and with loss of the p-arm of the X chromosome (50). However, in our study, ear problems did not differ among karyotypes. The low frequency of hearing problems detected in our study could be due to lack of sufficient awareness and underdiagnosis.

Ophthalmologic anomalies can be observed in about 63% of patients with TS (1). Strabismus, ptosis, amblyopia and color blindness are seen in decreasing order. In one review, strabismus was found to be as frequent as 33% and ptosis as 16% (51). Some studies reported that myopia can be associated with TS (2,7,51). Among our cohort, 74 patients were detected to have ocular problems at presentation. The most common pathology was strabismus (n=41), followed by myopia (n=16) and ptosis (n=8). We cannot make any comment on the prevalence of these disorders as there was no information in the records for the rest of the patients.

Females with TS are prone to some skin pathologies such as pigmented nevi, psoriasis, alopecia areata and keloid formation after surgical interventions (52). Pigmented nevi were reported to be present in 27% of patients regardless of the karyotype and to show an increase with growth hormone replacement (53,54). The frequency of these dermatological problems in our study was 21.8%. Presence of nevi was the most common one followed by psoriasis, vitiligo, alopecia, keloid and other skin problems. Since the frequency of nevi may show an increase with age, TS patients should be monitored for this manifestation.

Orthopedic evaluation in TS patients is of importance starting from infancy as they may have a high risk of congenital hip dislocation. In adolescence, the risk for developing scoliosis is as high as 10% (55,56). In our study, the frequency of scoliosis was 3%. Scoliosis was more prevalent in children older than 10 years compared to those aged 5-10 years and was not seen in patients younger than 5 years.

Estrogen deficiency in patients with TS increases the risk of osteoporosis. Bone mass reduction has been reported in 25% of women with TS (1). Osteoporosis and osteopenia were present in 63 (15.3%) and 49 (11.1%) of our patients, respectively and increased with age (p=0.001). The relatively low rate of osteoporosis/osteopenia in our series compared to the literature was attributed to the younger age of our patients. Karyotypes of patients with osteoporosis, osteopenia or other orthopedic/skeletal problems did not show any differences of statistical significance.

TS patients generally have normal intelligence except for mosaic karyotype with ring X chromosome. However, they have impaired non-verbal skills and low arithmetic skills. In our study, 291 patients were assessed for psychosocial problems and 39.1% were found to have psychosocial problems. Learning difficulties at school, as well as mental and emotional disorders (diagnosed by pediatric psychiatrists) were the most commonly encountered problems. These findings could be related to increased awareness of these problems as well as to inadequate adjustment to social environment. Only 11 out of 36 patients with ring chromosomes had been evaluated for psychological disorders, but there appeared to be no difference between patients with ring chromosomes and patients with other karyotypes regarding mental problems. As expected, emotional and learning problems increased with age (p=0.02), while no age difference was seen regarding mental disorders.

TS girls exhibit more metabolic risk factors and reduced beta cell function compared to age- and BMI-standard deviation score-matched girls (57). Waist circumference and subcutaneous adipose tissue measurements were higher in girls with TS compared to an age-matched healthy adolescent control group with a BMI of +1 standard deviation (58). In our study, 124 (17%) patients had metabolic problems. Among these patients, 69 were obese (9.5%) and 40 were overweight (5.5%). However, these figures are not high since, according to the Turkish Ministry of Health reports for 2010-2014, the prevalence of obesity in Turkish girls between 3-17 ages is 14.7 %.

Type 2 diabetes mellitus is reported to be more common in TS. Impaired glucose tolerance is seen at early ages and although obesity is a common problem in these patients, is not associated with either karyotype or BMI (58,59,60,61). A total of 122 patients were evaluated for any disorders of glucose metabolism. The results demonstrated that 24 patients (19.6%) had insulin resistance (5 of these patients had normal BMI), 16 patients (13.1%) had impaired fasting glucose (6 of these patients had normal BMI), 6 patients (4.9%) had glucose intolerance and 3 patients had type 2 diabetes. Of the 3 type 2 diabetes patients, 2 were obese and one had a normal BMI. Their ages were 11, 16 and 18 years. It has been emphasized that haploinsufficiency for unknown Xp genes increases the risk for diabetes mellitus and that an excess dosage of Xq genes compounds the risk (62). In our study, insulin resistance and glucose intolerance were more common in patients with isochromosome Xq. In the majority of these cases, the abnormalities in glucose metabolism were related to obesity.

The present study provides data on the demographic characteristics and frequency of associated problems in TS during childhood, based on the experience of Pediatric Endocrinology centers in Turkey. The findings indicate that some of the related problems are not being thoroughly searched in all patients. In those with complete evaluation, frequency of associated problems and their relationship with the karyotype groups are similar to that reported for other populations.

## Figures and Tables

**Table 1:**
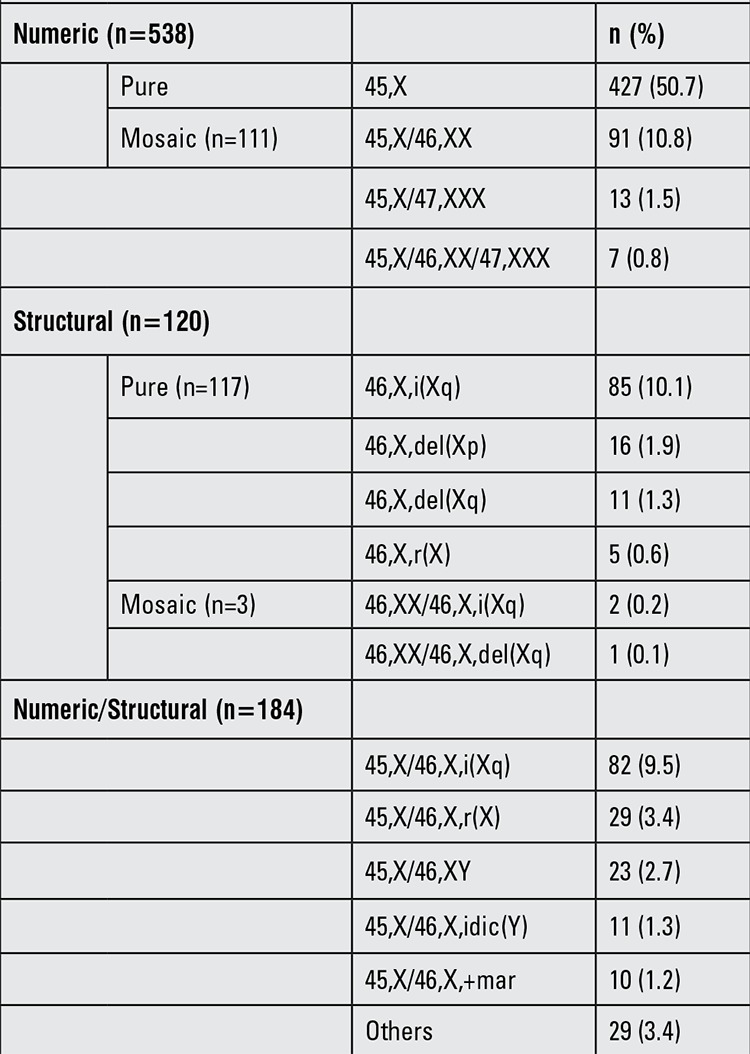
Distribution of the Turner syndrome patients by karyotype (n=842)

**Table 2:**
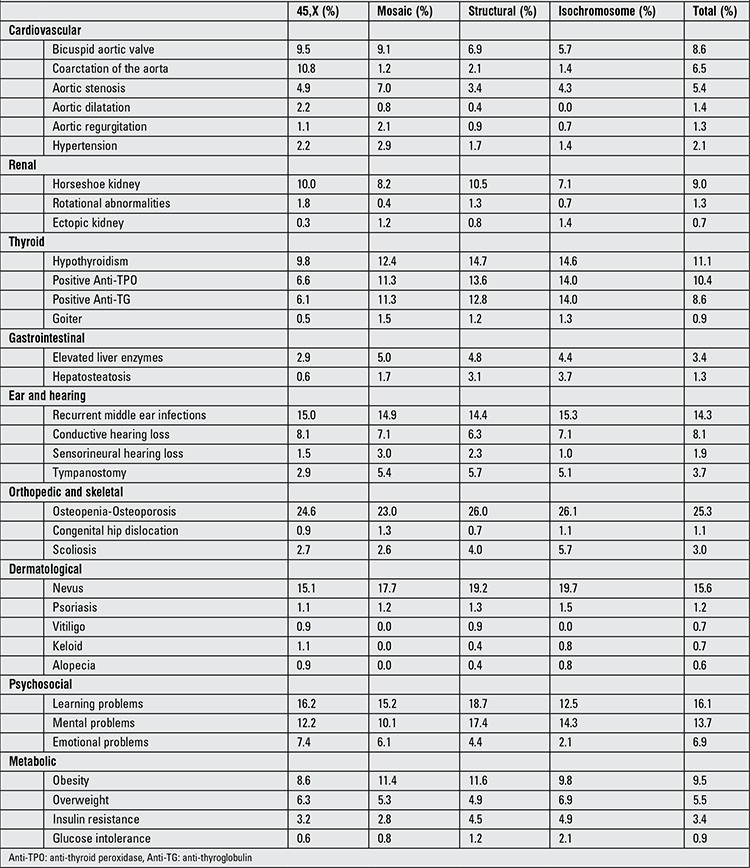
Distribution of pathologies in Turner syndrome patients by karyotype

**Table 3:**
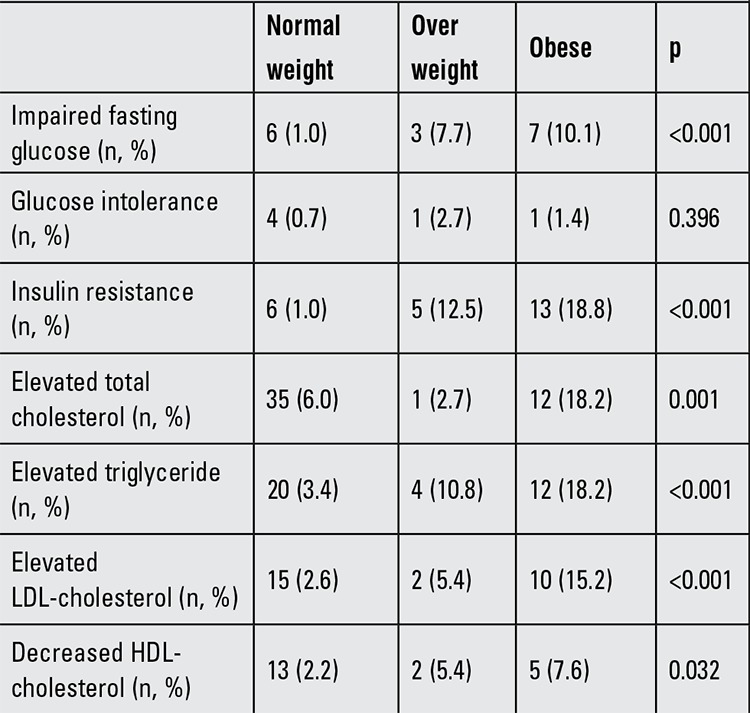
Some metabolic problems in Turner syndrome patients according to weight status
